# Educational films for improving screening and self-management of gestational diabetes in India and Uganda (GUIDES): study protocol for a cluster-randomised controlled trial

**DOI:** 10.1186/s13063-021-05435-x

**Published:** 2021-07-28

**Authors:** Laura L. Oakley, Deepa R, Arthur Namara, Biswamitra Sahu, Iliatha Papachristou Nadal, Yamuna Ana, Helen Coombe, Eugene Oteng-Ntim, Janet Seeley, Moffat Nyirenda, Giridhar Babu, Sanjay Kinra

**Affiliations:** 1 London School of Hygiene and Tropical Medicine, Keppel Street, London, WC1E 7HT UK; 2 Centre for Fertility and Health, Norwegian Institute of Public Health, Oslo, Norway; 3 Indian Institute of Public Health-Bangalore, Public Health Foundation of India (PHFI), Bangalore, India; 4 MRC/UVRI and LSHTM Uganda Research Unit, Entebbe, Uganda; 5 Department of Women’s Health, Guy’s and St Thomas’ NHS Foundation Trust, London, UK; 6 Medical Aid Films, London, UK

**Keywords:** Gestational diabetes, Cluster randomised controlled trial, India, Uganda

## Abstract

**Background:**

The prevalence of gestational diabetes mellitus (GDM) is rising rapidly in many low- and middle-income countries (LMICs). Most women with GDM in LMICs are undiagnosed and/or inadequately managed due to a lack of knowledge and skills about GDM on the part of both providers and patients. Following contextual analysis, we developed an educational/behavioural intervention for GDM delivered through a package of culturally tailored films. This trial aims to evaluate whether the intervention can improve the timely detection and management of GDM in two LMIC settings.

**Methods:**

Two independent cluster randomised controlled trials, one each to be conducted in Uganda and India. Thirty maternity facilities in each country have been recruited to the study and randomised in a 1:1 ratio to the intervention and control arms. The intervention comprises of three interconnected sets of films with the following aims: to improve knowledge of GDM guidelines and skills of health providers, to raise awareness of GDM screening among pregnant women and their families, and to improve confidence and skills in self-management among those diagnosed with GDM. In facilities randomised to the intervention arm, a GDM awareness-raising film will be shown in antenatal care waiting rooms, and four films for pregnant women with GDM will be shown in group settings and made available for viewing on mobile devices. Short films for doctors and nurses will be presented at professional development meetings. Data will be collected on approximately 10,000 pregnant women receiving care at participating facilities, with follow-up at 32 weeks gestational age and 6 weeks postnatally. Women who self-report a GDM diagnosis will be invited for a clinic visit at 34 weeks. Primary outcomes are (a) the proportion of women who report a GDM diagnosis by 32 weeks of pregnancy and (b) glycaemic control (fasting glucose and HbA1C) in women with GDM at ~34 weeks of pregnancy. The secondary outcome is a composite measure of GDM-related adverse perinatal-neonatal outcome.

**Discussion:**

Screening and management of GDM are suboptimal in most LMICs. We hypothesise that a scalable film-based intervention has the potential to improve the timely detection and management of GDM in varied LMIC settings.

**Trial registration:**

ClinicalTrials.gov NCT03937050, registered on 3 May 2019. Clinical Trials Registry India CTRI/2020/02/023605, registered on 26 February 2020.

## Background

Gestational diabetes mellitus (GDM) is associated with adverse perinatal outcomes and mortality [1–4]. Women with GDM have a ~50% greater risk of developing type 2 diabetes mellitus later in life, and the risk of obesity and diabetes among their offspring may also be increased [5, 6]. The prevalence of GDM is rising rapidly in many low and middle-income countries (LMICs), mirroring the rising prevalence of obesity and diabetes. In India, the age-standardised prevalence of GDM is estimated to be 28%, compared to 14% for high-income countries [7]. Population-based data for sub-Saharan Africa are sparse [8], but two recent hospital surveys from Uganda reported GDM prevalence estimates of 16% and 32%, respectively [9, 10]. The considerable healthcare utilisation costs of subsequent diabetes and premature cardiovascular disease in mother and offspring—and their impact on economic productivity—threatens the development agenda of LMICs [11].

Experience from high-income countries suggests that prevention of GDM, while desirable, is extremely challenging and resource-consuming [12, 13]. Timely detection and management of GDM may be a more cost-effective and feasible goal for resource-constrained settings and has been recommended as an early target for policy makers in LMICs [4, 14]. Despite this, most women with GDM in LMICs are undiagnosed and/or inadequately managed. A survey of key informants from 40 LMICs identified the unavailability of relevant guidelines and lack of knowledge about GDM on the part of both providers and patients as a significant barrier to detection and management of GDM [15, 16]. Limited availability and access to follow-up care, and low motivation and compliance of the patients, often linked to a lack of social support, were the other main factors identified. Where evidence-based GDM guidelines exist, they are often poorly applied in practice. In a national survey of doctors in India, which included specialist diabetologists and obstetricians, only a third were following the national guidelines for screening, of which two thirds could not recall the protocol correctly [17]. In another study conducted in Bangalore, India, only 50% of the doctors demonstrated good knowledge of GDM management [18].

Educational interventions aimed at improving knowledge of clinical guidelines have been shown to be effective in improving the practice of health providers and clinical outcomes, including diabetes [19, 20]. Women with GDM are more motivated to manage their condition due to its potential impact on the health of the fetus, yet structured programmes tailored for women with GDM are limited, even in high-income countries [21]. Evidence suggests that even highly effective interventions often fail to scale up in LMICs, if they are logistically challenging or require resources. Interventions designed to address chronic diseases in LMIC need to take into account specific structural and contextual factors which may influence efficacy and sustainability. Film-based interventions are easily scalable and adaptable, they can help to transcend literacy barriers and can be implemented with minimal infrastructure support. However, there is a paucity of evidence regarding the effective use of film in health behavioural interventions in LMIC settings [22].

**Fig. 1:**
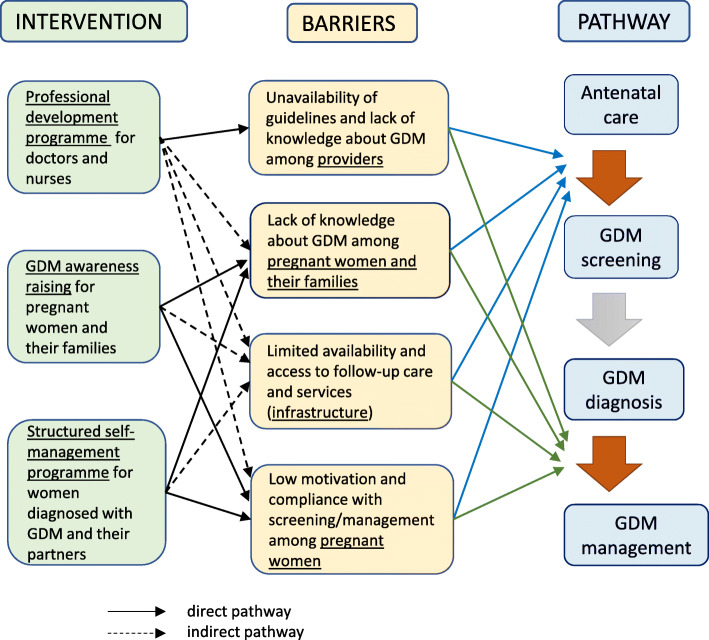
Hypothesised impact on barriers to GDM screening and management

We hypothesised that an educational/behavioural film-based intervention targeting knowledge, skills and behaviours at multiple levels—health providers, and pregnant women and their families—could improve detection and management of GDM in the short-term, and over a period of time could also impact on systemic barriers by increasing demand for better services, both within the public and growing private healthcare sectors in these countries (Fig. 1).

## Methods

### Aim

The primary aim of the two trials is to determine whether an educational/behavioural intervention delivered through a package of culturally tailored films for pregnant women, their family members and health providers can improve timely detection, glycaemic control and clinical outcomes of women with GDM.

### Design and setting

Two independent cluster-randomised controlled trials will be conducted, one in Uganda (Wakiso, Mpigi and Masaka) and one in India (Bengaluru). In Uganda, Wakiso, Mpigi and Masaka districts are a mix of urban/peri-urban areas and have relatively poor healthcare infrastructure and lower antenatal care attendance. Bengaluru, India is a densely populated urban area, with a relatively good infrastructure, and high antenatal care attendance. In India, current national guidelines recommend universal GDM screening using a single non-fasting glucose challenge test, with management following a structured pathway of a diet and activity programme followed by medical management where appropriate [23]. In Uganda, although summary recommendations regarding GDM screening and management are available [24], anecdotal evidence suggests that screening is largely non-existent and treatment is highly variable. As the two sites are at different stages of economic transition and have differing existing practise with regard to GDM screening and management, they will provide important complementary information about the generalizability of findings to other parts of sub-Saharan Africa and India.

### Participating health facilities

Government-funded health facilities providing maternity care and recording at least 200 deliveries a year were recruited as study sites. In Uganda, administrative clearance was sought from all participating facilities and District Health Offices. A total of 52 health facilities were assessed for participation. In India, consent was obtained from the Chief Health Officer (Clinical & Public Health) of the Bruhat Bengaluru Mahanagara Palike (Bengaluru, Karnataka), and 40 primary and secondary level health facilities were visited. Thirty facilities in each country were recruited to the trial, with an additional two facilities in Uganda providing participants for qualitative intervention development work. A list of participating facilities is available from the authors.

### Randomisation

Thirty health facilities in each country have been randomised in a 1:1 ratio to receive the intervention or control, with randomisation conducted separately for each country. Covariate constrained randomisation was performed by a statistician using the *cvcrand* command in Stata. Randomisation in India was restricted to ensure a good balance between the randomised arms with respect to the health facility (HF) level (levels I, II and III) and facility size (number of deliveries per year). For Uganda, the randomisation was restricted to ensure good balance with respect to HF level (level IV versus level III), facility and setting (rural versus non-rural).

### Participant recruitment

Consent to randomisation has been obtained at the cluster (health facility) level; individual-level informed consent will be sought for study procedures including data collection and blood tests. We plan to collect data on ~10,000 pregnant women in each trial. All pregnant women aged at least 18 years old and first attending for antenatal care <32 weeks at a participating health facility will be invited to participate in data collection. First antenatal contact ≥32 weeks will constitute an exclusion criterion as this is beyond the optimal gestational age for GDM screening. Local fieldworkers will visit clinics and screen women for eligibility. Consent to enter the study will be sought only after a full explanation has been given, an information leaflet offered and time allowed for consideration. Information sheets and consent forms will be available in appropriate languages (Luganda or English in Uganda; Kannada, Dhakani or English in India); consent discussions will be conducted in the appropriate language and a translator will be used if necessary. Additionally, for any qualitative data collection (interviews/focus groups), verbal consent will be obtained at the outset.

### Intervention

The film-based intervention evaluated in this trial was developed in conjunction with our partner, Medical Aid Films (https://www.medicalaidfilms.org/). Medical Aid Films are a UK-based charity focused on the use of innovative media to improve maternal and child health. They have a significant track record of producing high-quality films to support the training of health workers and community-focused behaviour change in LMICs. Working through an iterative co-production process, we developed a package of three interconnected educational/behavioural interventions aimed at (a) improving knowledge of GDM guidelines and skills among health providers involved in GDM management, (b) raising awareness of GDM and the importance of screening among pregnant women and their family members, and (c) improving confidence and skills in self-management for women diagnosed with GDM.

#### Development phase and theoretical background

The comprehensive intervention development phase included a desktop review, followed by a programme of focus group discussion and interviews with health professionals and pregnant women (with and without GDM) to understand the level of existing knowledge regarding GDM, skills, empowerment, resources, cultural beliefs, and aspirations [25]. Qualitative data were analysed using descriptive and interpretive phenomenology approaches. Subsequently, formative research workshops were held with key stakeholders in order to identify and review key messages. Drawing on the Health Belief Model (addressing barriers and emphasising perceived health benefits), film scripts were drafted and reviewed by a technical review committee. Filming on location was undertaken in January (India), February (Uganda) and September (India) 2020. Local women with experience of GDM were featured in the films, sharing their experiences of GDM. Alongside these narrative elements, we included excerpts from interviews with health care providers to reinforce key messages. Film content was piloted at rough-cut and subsequent editing stages with a small stakeholder group, before being finalised for implementation and evaluation in February 2021.

#### Intervention format

The format of the intervention is presented in Table 1. Films were produced in local languages (Lugunda for the Ugandan films; Dhakani and Kannada for the Indian films) with accompanying subtitles; English-language versions were also produced using a local-accented English-speaking voiceover artist. The duration of each of the films is between four and 10 minutes. Although the content of the films is broadly similar across both countries, the material has been adapted to reflect the cultural and clinical context. For example, dietary advice for pregnant women with GDM refers to popular and locally available food types. The Indian health professional films are aligned to national GDM guidelines, emphasizing universal GDM screening and home glucose monitoring for women with GDM. The Uganda versions reflect the more resource-limited setting, encouraging GDM screening and deferring to local guidelines regarding GDM management.

**Table 1:** Details of intervention components

Film package	Audience	Output	Focus	Delivery method	Country versions and available format
1	Doctors and nurses/midwives	1 short film for doctors, 1 short film for nurses	Knowledge and relevant clinical guidelines, skills for glucose monitoring and managing medication, and lifestyle counselling	Professional development meetings	2 films for India: -Kannada voiceover with subtitles -English voiceover with subtitles 2 films for Uganda: -English voiceover with subtitles
2	Pregnant women	1 short film	Raising awareness of GDM and the need for testing, healthy lifestyles during pregnancy	Screened in antenatal waiting rooms, available for mobile viewing	1 film for India: -Dhakani voiceover with subtitles -Kannada voiceover with subtitles 1 film for Uganda: -Luganda voiceover with subtitles -English voiceover with subtitles
3	Women diagnosed with GDM	4 short films (4 modules: basic physiology; skills to make lifestyle changes; medical and self-management; problem-solving and coping)	Education programme for women with GDM to empower them with the necessary knowledge, skills, and confidence to manage GDM successfully.	Screened during regular support sessions, available for mobile viewing	4 films for India: -Dhakani voiceover with subtitles -Kannada voiceover with subtitles 4 films for Uganda: -Luganda voiceover with subtitles -English voiceover with subtitles

#### Intervention delivery

The intervention has been designed with an emphasis on sustainability and scalability. In intervention arm facilities, films will be made available for viewing by doctors and nurses at regular professional meetings. Films for women and their families will be continuously screened in waiting areas of antenatal clinics (awareness-raising film) and during group education sessions for women diagnosed with GDM (films supporting GDM self-management). Where intervention settings do not have existing video/projection facilities, small low-cost projectors will be made available. Films for women will additionally be made available for viewing on mobile devices. Health facilities allocated to the control arm will follow usual care practices.

### Outcomes

The trial has two primary outcomes: (1) the proportion of women diagnosed with GDM by 32 weeks (self-reported) and (2) glycaemic control at ~34 weeks in women diagnosed with GDM (measured via fasting blood sugar and HbA1C). While both fasting blood sugar and HbA1C have some limitations as measures of glycaemic control in GDM patients, they offer the most pragmatic solution [26], particularly as glucose self-monitoring is not routinely available in all study settings so would itself constitute an intervention. We anticipate that any measurement error is likely be randomly distributed across the two trial arms.

The secondary outcome for the trial is a self-reported composite outcome consisting of individual indicators occurring during the perinatal and neonatal period which are associated with GDM: caesarean delivery, perinatal or neonatal death, or infant hospitalisation within 4 weeks of delivery. The use of such a composite variable is consistent with the approach taken in other GDM trials [27–29].

While two of the three study outcomes are self-reported, we hypothesise that these are unlikely to be misreported due to the nature of the outcomes and the contemporaneous nature of data collection. We will validate a sample of self-reported outcomes against hospital records where possible.

### Data collection

**Fig. 2:**
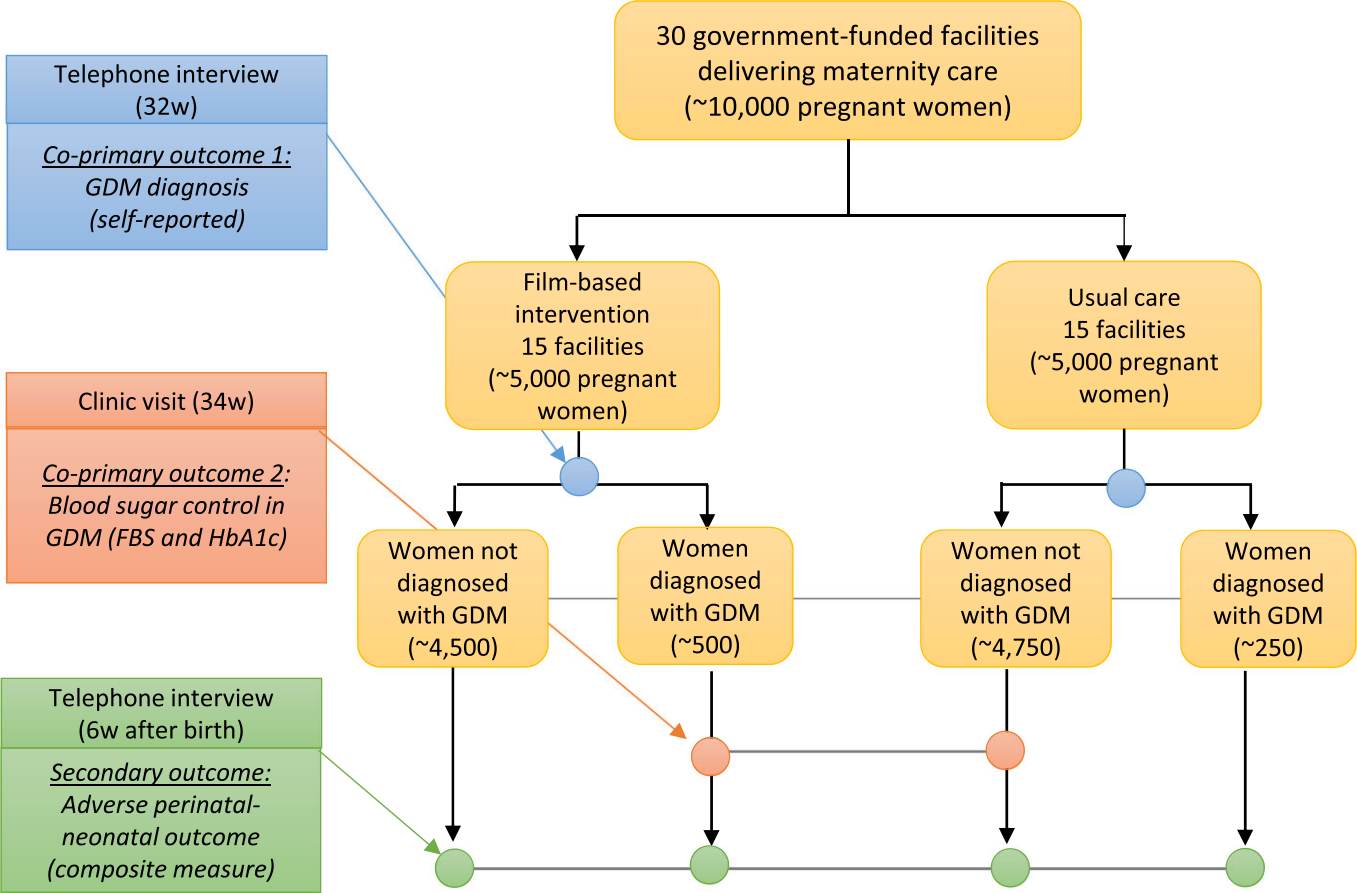
Study flow chart

Data will be collected from all participating women at three time points: baseline (after informed consent), 32 weeks of pregnancy and approximately 6 weeks after birth (Fig. 2). A description of the quantitative data to be collected at each time point is presented in Table 2. We will require a minimum 1-week interval between baseline and the first follow-up data collection. Baseline data will be collected via a face-to-face interview conducted in the antenatal care setting. Data collection at 32 weeks and postnatally will be via the telephone, or home visit if a telephone interview is not possible. The postnatal follow-up will be timed for approximately 6 weeks after the expected date of delivery, as final delivery dates will not be known by the fieldworkers.

**Table 2:** Schedule of data collection

Data collection point	Data type	Format	Measure
Baseline	Quantitative	Fieldworker-administered questionnaire (in person)	Sociodemographic Pregnancy history Current pregnancy GDM risk factors Tobacco and alcohol use Physical activity (IPAQ-SF) Dietary behaviour
32 weeks of pregnancy	Quantitative	Fieldworker-administered questionnaire (telephone)	GDM screening GDM diagnosisPregnancy complicationsKnowledge of GDM Physical activity (IPAQ-SF) Dietary behaviour Intervention recall (intervention arm only)
34 weeks of pregnancy (women with GDM only)	Biological sample	Clinic visit	Fasting Blood Sugar (FBS) and HbA1C
~6 weeks after birth	Quantitative	Fieldworker-administered questionnaire (telephone)	Perinatal and neonatal outcome

A fourth data collection point at ~34 weeks will be for women who report a GDM diagnosis at the 32-week telephone interview. These women (approximately 750 women in each country) will be invited to attend a clinic visit at ~34 weeks where fasting blood samples will be collected and tested for fasting blood glucose and HbA1c in order to assess co-primary outcome 1 (glycaemic control in women with GDM).

### Data management

Questionnaire data will be collected by fieldworkers and entered contemporaneously to a secure custom-designed app with in-built range and consistency checks. The app is designed with SSL encryption with SHA2, employs a role-based authentication system and follows the industry’s best practices for in-system security. It includes regular automated backup routines coupled with manually created periodic backup and point-in-time restoration. Data will be uploaded in real time to a secure password-protected cloud-based server, accessed only by authorised study staff. Fully anonymised data will be transferred for analysis using a secure encrypted data transfer service to investigators. Interview/focus group recordings will be transferred to a secure local server at the Uganda or India study coordinating centre. All staff involved in the study will be trained in the study procedures and good clinical practice.

### Data analysis

Available outcome data will be analysed, on an intention-to-treat basis, in mixed effects models, using a random effect term to account for clustering at the level of health facilities. We will consider adjustments for any baseline imbalances in covariates, and multiple imputations for missing data, if appropriate. For the co-primary outcome of glycaemic control, we recognise that the participants who are diagnosed with GDM in the two trial arms may not be comparable. We will consider the use of propensity score matching to take account of this non-randomised comparison, if appropriate. Effects of variation in exposure to the intervention and other relevant effect modifiers will be explored. Qualitative data will be transcribed and analysed using descriptive and interpretive phenomenology approaches. A social realist theoretical framework and thematic analysis will be used to reveal individual reactions in areas such as emotional engagement with content, acceptance of messaging, behavioural cues, and empathy and support.

### Process evaluation

A process evaluation will accompany the trial, drawing on the MRC framework to assess intervention delivery (fidelity, dose, reach). The process evaluation will help to clarify causal mechanisms, both those originally hypothesised (Fig. 1), and to identify unanticipated mechanisms. In a complex intervention of this kind, a process evaluation can help to explore the relationship between specific intervention components (each of the three film packages) and our trial outcomes. A range of quantitative and qualitative data will contribute to the process evaluation. Quantitative data will include random surveys of intervention sites assessing whether films are being shown as intended, and questionnaire data to measure whether women receiving care at intervention facilities recall viewing the study films. We will utilise ethnographic methods (direct observations including audio recordings of intervention delivery) and as well as structured interviews with doctors and nurses, pregnant women and their family members. We will use purposive sampling to recruit samples, provisionally planning for 5–15 ethnographic observations, and ~10–20 semi-structured interviews in each country. Quantitative data will be collected throughout the duration of the trial, and qualitative data collection will commence at 6 months.

### Power and sample size

Our pilot data suggests that we will be able to recruit ~10,000 pregnant women in each country during the 1-year recruitment period (~1 delivery/day/unit), of which ~10% (n=500) in intervention arm and ~5% (n=250) in the control arm are expected to be diagnosed with GDM (these figures may be lower in Uganda where screening prevalence is thought to be lower). Our estimated sample size requirements, based on preliminary data from Bengaluru and supplemented from literature, for 80% power at 5% significance level, and accounting for an indicative clustering of 0.01 for all outcomes, are 1218 pregnant women for GDM detection (5% vs 10%), 180 women with GDM for glycaemic control (fasting glucose difference of 0.3 mmol/L, for an SD of 0.9 mmol/L) [30] and 5935 women for our composite measure of adverse perinatal and neonatal outcome (30% vs 35%) [27]. These figures are considerably lower than our planned sample size.

### Ethical considerations and confidentiality

This study will be conducted according to the international standards of Good Clinical Practice (International Conference on Harmonization guidelines), Declaration of Helsinki, and International Ethical Guidelines for Biomedical Research Involving Human Subjects, applicable national government regulations, and institutional research policies and procedures. All investigators have received Good Clinical Practice training. Ethical approval has been obtained from the Uganda Virus Research Institute Research and Ethics Committee and the Uganda National Council of Science and Technology, The London School of Hygiene and Tropical Medicine’s Ethics Committee, and from the Indian Institute of Public Health, Institutional Ethics Committee, Bengaluru.

There may be minor discomfort from the taking of blood samples from women diagnosed with GDM; the patient information leaflet will warn women of this possibility. There is a possibility that the results of blood tests (fasting blood glucose) may necessitate additional clinical treatment/management. In this situation, the study team will communicate the test results to the usual healthcare provider. We do not anticipate any risks of the intervention itself.

We will anonymize all data, replacing identifiable information with study-specific identifiers at the earliest opportunity. Consent forms will be stored in a secure, locked location and access limited to the local PI and core project staff. Participants will be identified only by means of study numbers specific to each participant, and study databases will be password-protected. Upon request, participant records will be made available to the study sponsor.

For the qualitative data, when transcriptions are made, or accounts written up, the name of the respondent will be replaced by a participant number. All other names of individuals which may be mentioned by the participant will be replaced by initials.

### Trial governance and monitoring

A Trial Management Group (TMG) has been formed, consisting of the Chief Investigator, local Principal Investigators, local study coordinators and trial administrators. The TMG holds remote monthly meetings to oversee the day-to-day running of the trial. The TMG will be responsible for regular auditing of trial conduct and for communicating important protocol modifications to the wider project team and will also formulate key policies and working groups. A Trial Steering Committee (TSC) has been convened to provide overall supervision of both trials, and will meet remotely at regular intervals determined by need, at a minimum of once a year. The TSC is chaired by an independent member and includes experts in the field of maternal medicine, health psychology and clinical trials. We do not anticipate any adverse events or unintended effects of this educational/behavioural intervention, nor do we envisage the need for any specific post-trial care as a result of trial participation. A Data and Safety Monitoring Board (DSMB) at the Indian Institute of Public Health Bengaluru has responsibility for regular monitoring of the trial in India. In Uganda, a Data Monitoring Committee (DMC) has been convened. Both the India DSMB and Uganda DMC are chaired by independent members and meet as needed, at a minimum of once a year. Charters and Terms of Reference for the TSC, DSMB and DMC are available on request. The study sponsor is the London School of Hygiene and Tropical Medicine.

### Dissemination plans

We will publish results arising from the trial including qualitative work as well as the primary trial analyses, in established peer-reviewed journals complying with CONSORT and other relevant guidance. We will also disseminate the results at international conferences and meetings. All publications and presentations relating to the study will be authorised by the Trial Management Group. In the final phase of the study, we will produce a research summary and policy recommendations, shared through our study website, newsletter and professional networks. We will organise dissemination meetings with national policy-makers (Ministry of Health, Uganda; Ministry of Health and Family Welfare, India). A lay summary of our research will be disseminated to stakeholders via social media, local networks and participating health facilities.

## Discussion

This trial will evaluate whether an educational/behavioural intervention delivered through a package of culturally tailored films can provide scalable improvements in the timely detection and management of GDM.

Findings from this project will contribute to scientific evidence underpinning the use of films in cost-effectively scaling up behavioural interventions in LMIC settings. In controlled trials in HIC settings, films have shown to be effective in increasing participation in HIV and prostate cancer screening, improving self-care in heart failure patients, stroke literacy and treatment adherence [20, 31–33]. Visual information is processed far more efficiently than text [34], making it an effective method of communication, especially in low-literacy settings where populations have limited opportunity to develop text-based processing skills. Films are an exceedingly simple and scalable intervention that requires minimal infrastructure. They can be shown via low-cost projectors in high-volume settings (such as waiting rooms) and increasingly can be disseminated for viewing via mobile devices such as smartphones. Films can be used to deliver simple and accessible information, and consistent messages [31], yet also provoke discussion and challenge existing beliefs and stigma to promote behaviour change. Robust data on the effectiveness of films in delivering behavioural interventions in LMICs is critically needed, as given their scalability, they have the potential to become a key component of strategies to support behaviour change in chronic diseases. Additionally, this trial will contribute to evidence on culturally tailored self-management interventions for long-term conditions, particularly in LMICs, for which existing evidence is limited.

### Trial status

At the time of submission, health facilities have been recruited and randomised, the film-based intervention finalised and participant recruitment will commence shortly. Protocol Version 3.2 (26 November 2020).

## Data Availability

Not applicable.

## Abbreviations

DMC: Data Monitoring Committee
DSMB: Data Monitoring and Safety Board
GDM: Gestational diabetes mellitus
GUIDES: Gestational diabetes in Uganda and India: Design and Evaluation of Educational Films for improving Screening and Self-management
LMIC: Low- and middle-income countries
LSHTM: London School of Hygiene and Tropical Medicine
MAF: Medical Aid Films
MRC: Medical Research Council
RCT: Randomised controlled trial
TSC: Trial Steering Committee
